# A nerve conduit filled with Wnt5a‐loaded fibrin hydrogels promotes peripheral nerve regeneration

**DOI:** 10.1111/cns.13752

**Published:** 2021-11-02

**Authors:** Yi‐jun Liu, Xiao‐feng Chen, Li‐ping Zhou, Feng Rao, Dian‐ying Zhang, Yan‐hua Wang

**Affiliations:** ^1^ Department of Orthopedics and Trauma Peking University People’s Hospital Beijing China; ^2^ Department of Foot and Ankle Surgery Center for Orthopaedic Surgery the Third Affiliated Hospital of Southern Medical University Guangzhou China; ^3^ Department of Orthopedics and Trauma Key Laboratory of Trauma and Neural Regeneration (Ministry of Education/ Peking University) Beijing China; ^4^ Beijing Key Laboratory for Bioengineering and Sensing Technology Daxing Research Institute School of Chemistry & Biological Engineering University of Science & Technology Beijing Beijing China; ^5^ Trauma Medicine Center Peking University People’s Hospital Beijing China

**Keywords:** nerve conduit, nerve growth factor, peripheral nerve injury, vascular endothelial growth factor, Wnt5a

## Abstract

**Aims:**

Peripheral nerve injury is a significant clinical problem with a substantial impact on quality of life, for which no optimal treatment has been found. This study aimed to analyze the effect and mechanism of Wnt5a‐loaded fibrin hydrogel on a 10‐mm rat sciatic nerve defect.

**Methods:**

The Wnt5a‐loaded fibrin hydrogel was synthesized by mixing a Wnt5a solution with thrombin and fibrinogen solutions. The loading capacity and release profile of Wnt5a‐loaded fibrin hydrogel and the effect of Wnt5a on Schwann cells were evaluated in vitro. We also assessed the in vivo repair status via histological analysis of the regenerative nerve and gastrocnemius muscle, electrophysiological examination, gait analysis, and muscle wet weight.

**Results:**

We developed a nerve conduit filled with Wnt5a‐loaded fibrin hydrogel (Fn) as a sustained‐release system to repair a 10‐mm rat sciatic nerve defect. In vitro, Wnt5a could promote SC proliferation and the gene expression of vascular endothelial growth factor (VEGF), nerve growth factor (NGF), and cholinergic neurotrophic factor (CNTF), as well as the protein secretion of VEGF and NGF. In vivo, the Wnt5a/Fn group was superior to the hollow, fibrin hydrogel, and Wnt5a groups in terms of axonal growth, myelination, electrophysiological recovery, target organ innervation, and motor function recovery 12 weeks after the operation.

**Conclusion:**

The Wnt5a/Fn nerve conduit can promote peripheral nerve defect regeneration, with potential clinical applications. The mechanism for this may be the facilitation of multiple neurotrophin secretion, combining vascularization and neurotrophic growth cues.

## Graphical abstract

The nerve conduit filled with Wnt5a‐loaded fibrin hydrogel as a sustained‐release system was developed to repair a 10‐mm rat sciatic nerve defect, which can promote peripheral nerve defect regeneration with potential in clinical application. The mechanism may be the facilitation of multiple neurotrophin secretion combining vascularization and neurotrophic growth cues.

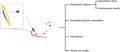

## 1 INTRODUCTION

Peripheral nerve injury is a global clinical problem that can cause impaired limb function and poses a heavy burden on society.^1^, ^2^ 5% of trauma patients may experience sequelae such as motor, sensory, and autonomic dysfunction due to peripheral nerve injury, which can seriously affect the quality of life.^3^

Treatment by autologous nerve transplantation is the gold standard for peripheral nerve defects. However, the clinical application of autografts is restricted due to the limited donor area, dysfunction of donor sites, and the need for additional surgery for autologous nerve collection.^4^ Neural tissue engineering can help avoid these disadvantages and has the potential to achieve an efficacy similar to that of autologous nerve transplantation. Thus, neural scaffolds have been the focus of current research on the peripheral nerve gap. Improvement of the regenerative microenvironment is a critical aspect of neural tissue engineering, such as signaling molecules and neurotrophic factors (NTFs).^1^, ^2^

The proteins from the wingless‐type murine mammary tumor virus integration site (Wnt) family regulate many cellular and physiological processes, such as cell fate, proliferation, migration, and differentiation.^5^ Wnt5a, which is a noncanonical Wnt member, plays a role in the nervous system^6^ that may be crucial for peripheral nerve regeneration for the following reasons: First, Wnt5a can promote growth cone and axon growth by stimulating calpain activity in the central nervous system.^7^ Second, Wnt5a can promote axonal differentiation by activating the protein kinase C.^8^ Third, Wnt5a is a key downstream protein in nerve growth factor (NGF) signaling; it promotes axonal branching and extension in the sympathetic nervous system.^9^ Fourth, Wnt5a can regulate Schwann cell (SC) regeneration and proliferation via the Wnt/c‐Jun and phosphatase and tensin homologue deleted on chromosome 10 (PTEN) pathways, negatively affecting when silenced by the lentiviral vector.^10^ Fifth, Wnt5a signaling is crucial for pathological angiogenesis, especially in the inflammatory area.^11^, ^12^ Early vasculature formation is important in guiding SC migration and axon extension during peripheral nerve regeneration.^13^ However, although Wnt5a is closely associated with the nervous system, its repair effect on peripheral nerve injury remains unclear.

Fibrin hydrogel is synthesized by thrombin and fibrinogen and is widely used as a lumen filler for neural scaffolds because of its good biocompatibility, degradability, and porous structures.^14^, ^15^, ^16^ Thus, we used fibrin hydrogel as a sustained‐release vehicle for the delivery system of Wnt5a.

In this study, we fabricated a nerve conduit filled with a Wnt5a‐loaded fibrin hydrogel and characterized its microstructure, loading capacity, and release profile. We also investigated the effect of Wnt5a on the SCs in vitro and the repair effect of a Wnt5a‐loaded fibrin hydrogel conduit on a 10‐mm rat sciatic nerve defect in vivo. The purpose of this study was to evaluate the repair effect and mechanism of Wnt5a‐loaded fibrin hydrogel on peripheral nerve regeneration.

## 2 MATERIALS AND METHODS

### 2.1 Synthesis of Wnt5a‐loaded fibrin hydrogel

The chitosan nerve conduit (CNC) was fabricated based on a previous protocol (National Invention Patent No. ZL01136314.2).^17^ We mixed 100 UI/mL thrombin (T4648, Sigma‐Aldrich) and 0.8% fibrinogen (f8630, Sigma‐Aldrich, T4648, Sigma‐Aldrich) solutions at a ratio of 1:4 to form the fibrin hydrogel.^15^, ^18^ We created a Wnt5a‐loaded fibrin hydrogel by mixing the Wnt5a solution (7815‐NG, R&D Systems) in thrombin and fibrinogen solutions, respectively, before the fibrin hydrogel was synthesized. As suggested,^9^ the final concentration of Wnt5a was 100 ng/mL.

### 2.2 Scanning electron microscopy (SEM)

The fibrin hydrogel was fixed with 2.5% glutaraldehyde for 30 min, dehydrated in graded concentrations of ethanol (30%, 50%, 70%, 90%, 95%, and 100%), and subsequently dried at −80°C in a freeze dryer. The samples were sputter‐coated, cross sectioned with gold, and then examined by SEM (6700F field‐emission SEM; JEOL).^15^

### 2.3 Loading capacity and release profile of Wnt5a‐loaded fibrin hydrogel

The absorbance of Wnt5a in the fibrin hydrogel was measured by ultraviolet visible near‐infrared spectrophotometer (UV–vis–NIR, Shimadzu UV‐2450), when the mass ratio (w/w) of Wnt5a and the fibrin hydrogel was 1.2, 1.4, 1.6, and 1.8, respectively. The loading capacity (w/w%) was calculated based on the absorbance.^19^ At specific time points (1, 3, 7, 12, 14, 21, 28, and 35 days), we tested the upper layer solution and subsequently added an equal amount of phosphate buffer saline solution (2 µL). The amount of Wnt5a in the samples was detected via UV‐via‐NIR spectroscopy.

### 2.4 Isolation and culture of Schwann cells

The SCs were harvested and purified from 3‐day‐old neonatal rats as reported previously.^15^, ^20^ Briefly, the sciatic nerves of the neonatal rats were harvested after disinfection. The sciatic nerve was cut into 1‐mm segments, placed in 0.2% collagenase NB4 (Nordmark Arzneimittel GmbH&C) for 20 min at 37°C, and resuspended in Dulbecco's modified Eagle's medium and nutrient mixture F‐12 (DMEM/F‐12, Thermo Fisher) containing 10% fetal bovine serum medium (Thermo Fisher). The SCs were cultured in DMEM/F‐12 containing 10% FBS, 2 mM forskolin, and 10 ng/mL epidermal growth factor receptor (R&D). The SCs were seeded into 96‐well or 6‐well plates after hydrogel formation at a density of 1 × 10^5^ cells/mL in each group.

### 2.5 Cell Counting Kit‐8 (CCK‐8)

The SCs were divided into the control group and the Wnt5a group, seeded into 96‐well plates after hydrogel formation at a density of 1 × 105 cells/mL, and cultured for 24 and 48 h. We incubated the cells with a 10 μl CCK‐8 solution (C0040, Beyotime) for 4 h. The absorbance at 450 nm was measured using a microplate reader (BMG Labtech). The CCK‐8 results were shown using A_experimental_‐A_blank_.^21^

### 2.6 Quantitative real‐time polymerase chain reaction (qRT‐PCR)

After the SCs were cultured for 5 days, the total ribonucleic acid (RNA) was extracted with TRIzol (Thermo Fisher), and the purity and concentration of the RNA sample solution were determined using a NanoPhotometer^®^ (IMPLEN). RNA solutions with an optical density 260/280 value between 1.8 and 2.0 were used for qRT‐PCR. We used a 5X All‐In‐One RT MasterMix Kit (QPK‐201, Toyobo Life Science) for reverse transcription into cDNA and SYBR Green (G592, ABM) for qRT‐PCR. The primer sequences are listed in Table 1. The relative expression of the target genes was normalized according to glyceraldehyde 3‐phosphate dehydrogenase (GADPH).^20^

**TABLE 1:** The primer sequences for qRT‐PCR

Primers	Forward	Reverse
VEGF	AACTTCTACCCGTGCCTT	ACTTAGGTCAGCGTTTCC
NGF	CAGTGTCAGTGTGTGGGTTGGA	GGCTCGGCACTTGGTCTCAA
CNTF	GCCATTCGCTCATACCTCTGTC	TGTGGGCCAACCCTACTTGATG
GADPH	ATGGTGAAGGTCGGTGTGAACG	TTACTCCTTGGAGGCCATGTAG

Abbreviations: CNTF, cholinergic neurotrophic factor; GADPH, glyceraldehyde 3‐phosphate dehydrogenase; NGF, nerve growth factor; qRT‐PCR, quantitative real‐time polymerase chain reaction; VEGF, vascular endothelial growth factor.

### 2.7 Enzyme‐linked immunosorbent assay (ELISA)

The culture supernatant was collected. The concentrations of the vascular endothelial growth factor (VEGF), NGF, and cholinergic neurotrophic factor (CNTF) were measured using an enzyme‐linked immunosorbent assay (Jiangsu, Meimian Industrial Co., Ltd.).

### 2.8 Animals

Thirty female Sprague‐Dawley rats weighing 200–220 g were used for this experiment (Beijing Charles River Company) and were kept in specified pathogen‐free conditions and randomly divided into five groups using random number table method (*n* = 5). Group 1 received a hollow nerve conduit (hollow group), Group 2 received a nerve conduit filled with fibrin hydrogel (Fn group), Group 3 received a nerve conduit filled with Wnt5a solution at a concentration of 100 ng/mL (Wnt5a group), Group 4 received a nerve conduit filled with Wnt5a‐loaded fibrin hydrogel (Wnt5a/Fn group), and Group 5 received autologous nerve transplantation (autograft group). The rats were kept in the animal experiment center of our institution, with a room temperature of 20–22°C. The rats were provided a reasonable diet and a 12/12‐hour night/dark cycle. This study was approved by the Animal Ethics Review Committee of the Peking University People's Hospital (approval number: 2018PHC020). The animal data reporting has followed the ARRIVE guidelines.^22^

### 2.9 Surgical procedures

The specific steps for constructing the sciatic nerve defect model of SD rats were as follows^20^, ^23^: The SD rats were anesthetized with 5% isoflurane and maintained with 1.5–2% isoflurane. The rats were then fixed in a prone position, and the right sciatic nerve was exposed by blunt separation. The sciatic nerve was cut from the lower edge of the piriformis muscle to create a 10‐mm defect. In the hollow, Fn, Wnt5a, and Wnt5a/Fn groups, the nerve conduit was micro‐sutured into the sciatic nerve with a 10–0 nylon suture under a microscope; a 12‐mm nerve conduit was used to repair the nerve gap. In the autograft group, a 10‐mm section of the sciatic nerve was cut off, turned 180°, and sutured through the epineurium. Finally, the incision was sutured layer‐by‐layer with 4–0 sutures.

### 2.10 Gait analysis

Gait analysis was used to evaluate the recovery of motor function.^20^, ^24^ Before the experiment, the SD rats were placed on the CatWalk XT machine (Noldus) for training to have consecutive uninterrupted runs.^21^ Twelve weeks after the operation, the motor function of the SD rats was evaluated using the CatWalk XT 10.6 gait analysis system (Noldus). The walking tracks and footprints were recorded for the gait analysis. The sciatic functional index (SFI) was calculated using the following formula: SFI =−38.3 [(EPL‐NPL)/NPL] +109.5 [(ETS‐NTS)/NTS) +13.3 [(EITS‐NITS)/NITS] −8.8.^15^ Here, EPL is the distance between the heel and the third toe on the injured side, and NPL is the distance between the heel and the third toe on the normal side. The EITS is the distance between the second and fourth toes on the injured side. The NITS is the distance between the second and fourth toes on the normal side.

### 2.11 Electrophysiological examination

Twelve weeks after the operation, the animals were anesthetized with 5% isoflurane, and the sciatic nerve and gastrocnemius were exposed for electrophysiological testing using an electrophysiological instrument (Keypoint, Nørresundby, Denmark). Stimulation electrodes were placed on the proximal and distal ends of the nerve grafts (3 mA, 1 Hz). Two recording electrodes were inserted into the gastrocnemius muscle, and ground electrodes were placed on the ipsilateral gluteus maximus. The latency and peak value of the compound muscle action potential (CMAP) were recorded and analyzed.

### 2.12 Muscle wet weight and histological analysis

The gastrocnemius was then completely stripped and weighed. The wet weight ratio of gastrocnemius was calculated by the wet weight of the injured side to the wet weight of the normal side. After weighing, the gastrocnemius muscle was immersed in 4% paraformaldehyde overnight, washed, dehydrated in graded ethanol, and subsequently embedded in paraffin wax. The muscle was cross‐cut into 5‐μm‐thick sections for Masson staining. A microscope (Leica, MZ75) was used to record the images. The ImageJ software (Media Cybernetics Inc.) was used to calculate the cross‐sectional area of the muscle fibers.

### 2.13 Morphometric analysis of regenerated axons

Twelve weeks after the operation, the distal end of the regenerated nerve segment was first fixed in 2.5% glutaraldehyde for 6 h and then immersed using 1% osmium tetroxide (pH 7.3, 4°C, 2 h). The tissue was embedded in resin and cross‐cut into slices with a thickness of 1.0 μm and 50.0 nm after dehydration. The sections with a thickness of 1.0 μm were stained with toluidine blue and photographed under an optical microscope (AH3; Olympus) to calculate the total number of myelin axons per square millimeter. The sections with a thickness of 50.0 nm were stained with uranyl acetate and lead citrate and photographed under a transmission electron microscope (TEM, TecnaiTM g2, FEI, NL). The ImageJ software was used to calculate the thickness of the myelin sheath, axon diameter, and density of the myelinated nerve fibers.

### 2.14 Immunofluorescence

Twelve weeks after surgery, the regenerated sciatic nerve was harvested, fixed with 4% paraformaldehyde, and dehydrated with 30% sucrose solution. A 12‐μm‐thick cross section of the nerve tissue was obtained using a cryostat (Leica). Immunofluorescence analysis was performed on the middle part of the regenerated nerves using S100 (S2644; Sigma‐Aldrich), NF200 (N0142; Sigma‐Aldrich), and CD31 antibody (ab182981; Abcam). The primary antibody was incubated overnight at 4°C. The sections were stained using mixed second antibodies, Alexa Fluor 594 and Alexa Fluor 488 (ab182981, ab150084, Abcam), for 1 h at room temperature and subsequently incubated with DAPI (C0060, Solarbio) for 10 min. Finally, the samples were observed using a confocal laser scanning microscope (LSM710, Leica).^20^, ^25^ The microvessel density per cross section was calculated using the ImageJ software.^26^

### 2.15 Statistics

Statistical analysis was performed using the SPSS 23.0 software (SPSS Inc.). The Shapiro‐Wilk test was used to test the normality. Quantitative data with a normal distribution were shown as mean ±standard deviation, and those with a non‐normal distribution were shown as medians (interquartile range, IQR). Student's *t*‐test and one‐way analysis of variance (ANOVA) were used when data were normally distributed and group size was equal to or greater than 10. Mann‐Whitney *U* or Kruskal‐Wallis one‐way ANOVA test was applied in the case of non‐normal distribution. Statistical significance was set at *p* < 0.05.

## 3 RESULTS

### 3.1 Characteristics of Wnt5a‐loaded fibrin hydrogel

**FIGURE 1:**
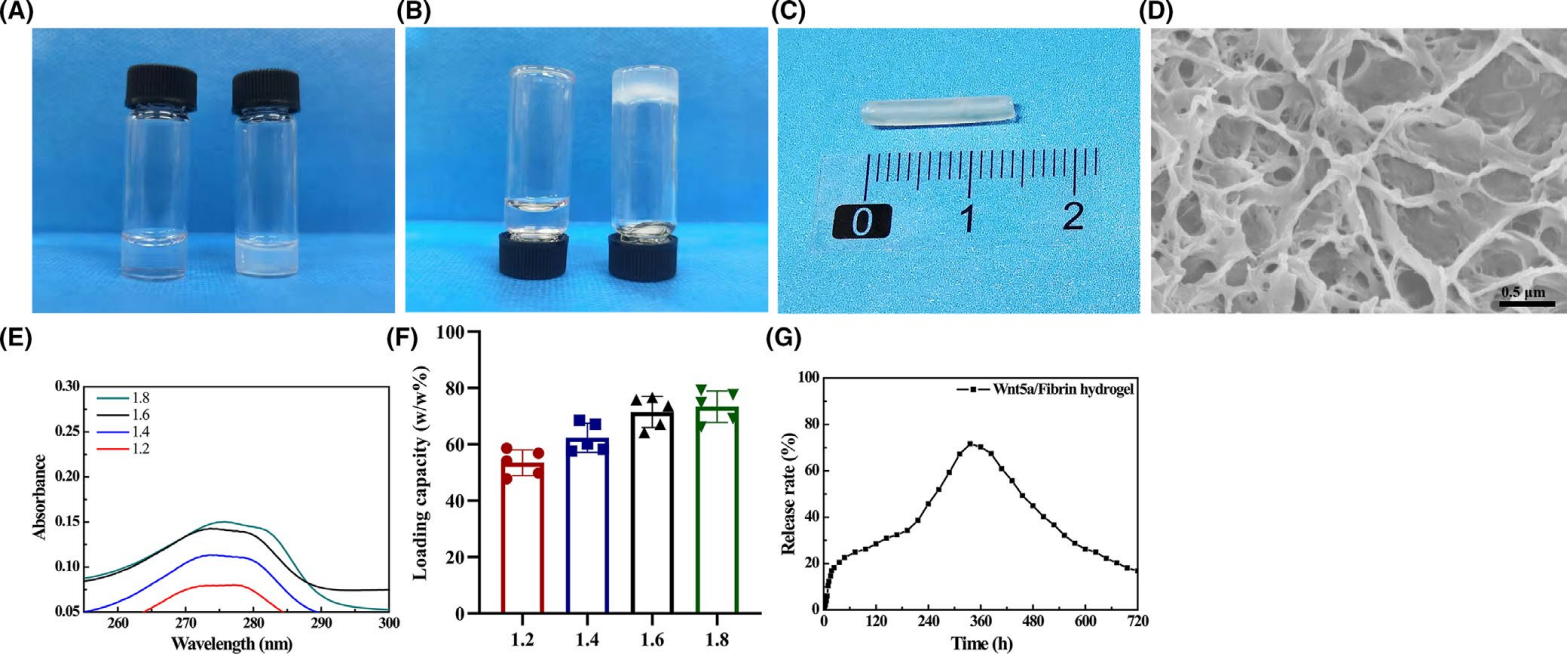
Characterization of the Wnt5a‐loaded fibrin hydrogel. (A–B) Gross view of the saline solution (left) and fibrin hydrogel (right) at upright and upside‐down position. (C) A representative photograph of the nerve conduit filled with the Wnt5a‐loaded fibrin hydrogel. (D) Representative SEM images. (E) The absorbance of Wnt5a when the mass ratio (w/w) of Wnt5a and fibrin hydrogel was 1.2, 1.4, 1.6, and 1.8, respectively. (F) The loading capacity (w/w%) of Wnt5a‐loaded fibrin hydrogel in each group. (G) The sustained‐release profile of Wnt5a‐loaded fibrin hydrogel

The gross view of the saline solution and fibrin hydrogel is shown in Figure 1A,B, indicating that the fibrin hydrogel was in a gel state. A representative photograph of the nerve conduit filled with the Wnt5a‐loaded fibrin hydrogel is shown in Figure 1C. SEM indicated that the fibrin hydrogel was a 3D porous structure with interconnected pores (Figure 1D). The UV‐vis‐NIR absorbance was almost the same between the two groups with 1.6 and 1.8 mass ratios (w/w), indicating that the fibrin hydrogel reached a saturation state at the mass ratio of 1.6 (w/w) (Figure 1E). The loading capacity (w/w%) of fibrin hydrogel for Wnt5a was 73.7% in the saturated state (Figure 1F). The sustained‐release profile of Wnt5a‐loaded fibrin hydrogel indicated that 20% of Wnt5a was rapidly released in the first 1.5 days, and the release rate slowly increased to a peak at 14 days (71.6%) (Figure 1G).

### 3.2 Wnt5a promotes neurotrophin secretion

**FIGURE 2:**
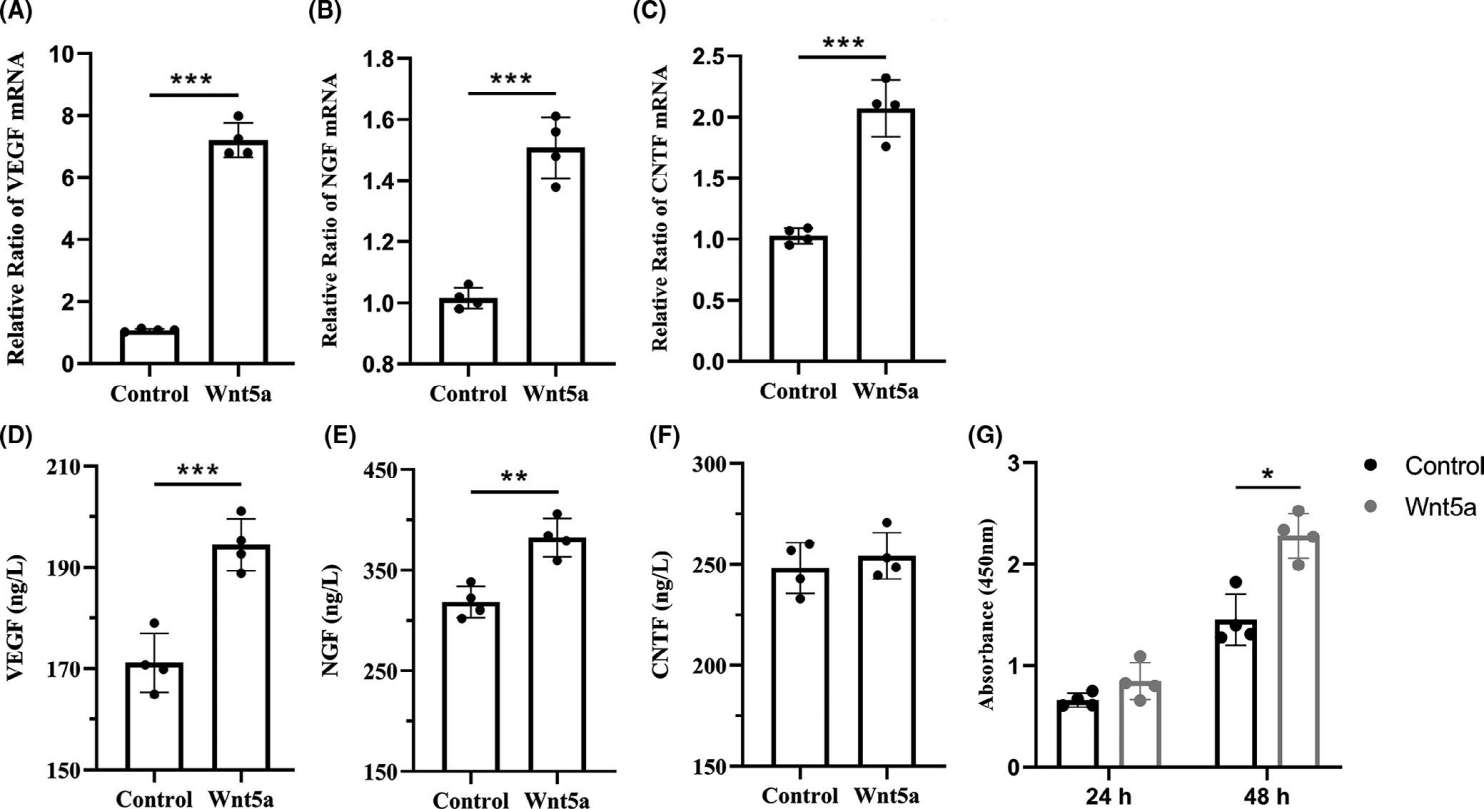
Wnt5a regulates SC proliferation and secretion. (A–C) qRT‐PCR results showed relative expression levels of VEGF, NGF, and CNTF mRNA. (D–F) ELISA results indicated the concentrations of VEGF, NGF, and CNTF. (G) CCK‐8 results showed the effect of Wnt5a on SC proliferation after incubation for 24 h and 48 h.*p < 0.05, **p < 0.01, ***p < 0.001. Data are expressed as the mean ±SD. SCs, Schwann cells; VEGF, vascular endothelial growth factor; NGF, nerve growth factor; CNTF, cholinergic neurotrophic factor. qRT‐PCR, quantitative real‐time polymerase chain reaction

The relative expression levels of VEGF, NGF, and CNTF mRNA were significantly higher in the Wnt5a group (*p* < 0.05), with a value 6.7 ± 0.4‐fold, 1.5 ± 0.1‐fold, and 2.0 ± 0.1‐fold of that of the control group, respectively (Figure 2A–C). The concentrations of VEGF (194.2 ± 4.4 ng/L) and NGF (382.3 ± 16.5 ng/L) were significantly higher in the Wnt5a group than in the control group (171.1 ± 5.1 ng/L, 318.4 ± 13.7 ng/L) (*p* < 0.05) (Figure 2D–F). There was no significant difference in the concentration of CNTF between the two groups.

### 3.3 Wnt5a promotes SC proliferation

After 24 h of Wnt5a incubation, the OD value had an increasing trend compared with that of the control group (*p* > 0.05). When the SCs were cultured for 48 h, the OD value of the Wnt5a group (2.3 ± 0.19) was significantly higher than that of the control group (1.5 ± 0.2) (Figure 2G).

### 3.4 Electrophysiological recovery

**FIGURE 3:**
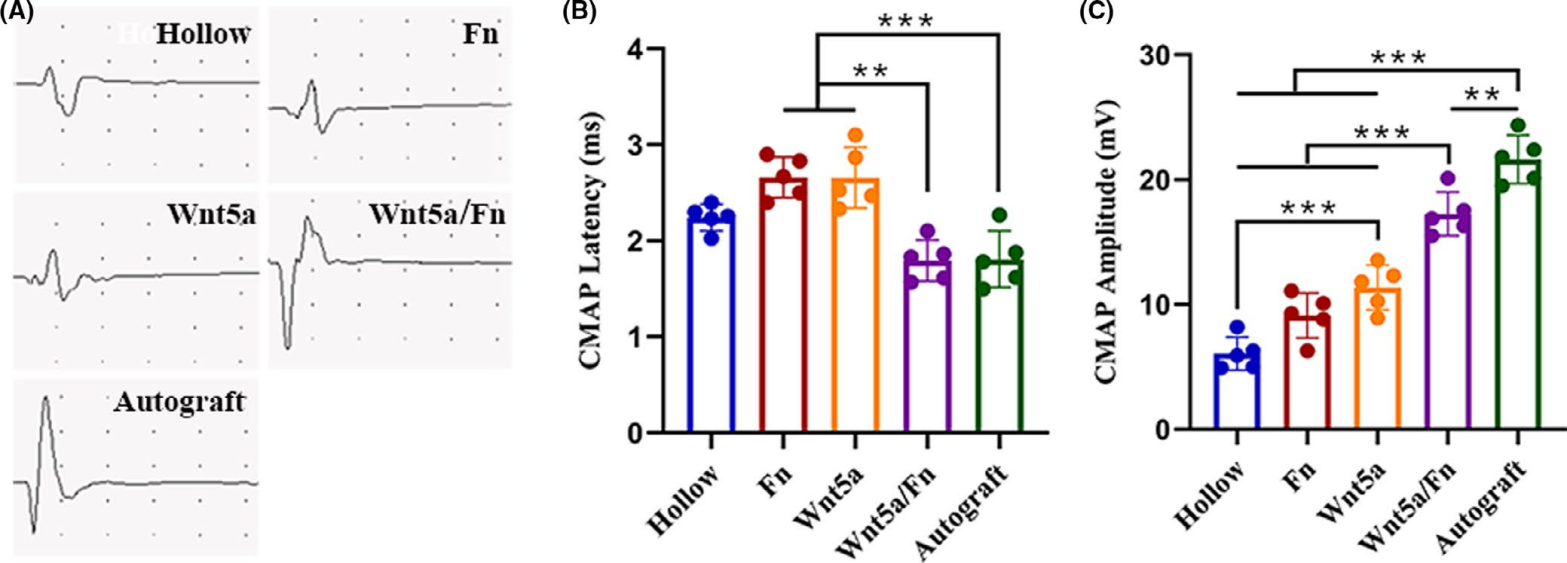
Electrophysiological examination of each group 12 weeks after the operation. (A) Representative images of the CMAPs of each group. (B) CMAP latency of each group. (C) CMAP amplitude of each group. **p < 0.01, ***p < 0.001. Data are expressed as the mean ±SD. CMAP, compound muscle action potential. Fn, fibrin hydrogel group

Figure 3A shows the representative CMAP oscillograms at 12 weeks after surgery. The CMAP latency of the Wnt5a/Fn and autograft groups was significantly lower than those of the Fn and Wnt5a groups (*p* < 0.05); however, there was no significant difference in the CMAP latency in the hollow group (Figure 3B). The CMAP amplitudes of the Wnt5a/Fn and autograft groups were significantly greater than those in the hollow, Fn, and Wnt5a groups (*p* < 0.05). Moreover, the CMAP amplitude of the autograft group was significantly greater than that of the Wnt5a/Fn group (*p* < 0.05; Figure 3C).

### 3.5 Axonal growth and myelination

**FIGURE 4:**
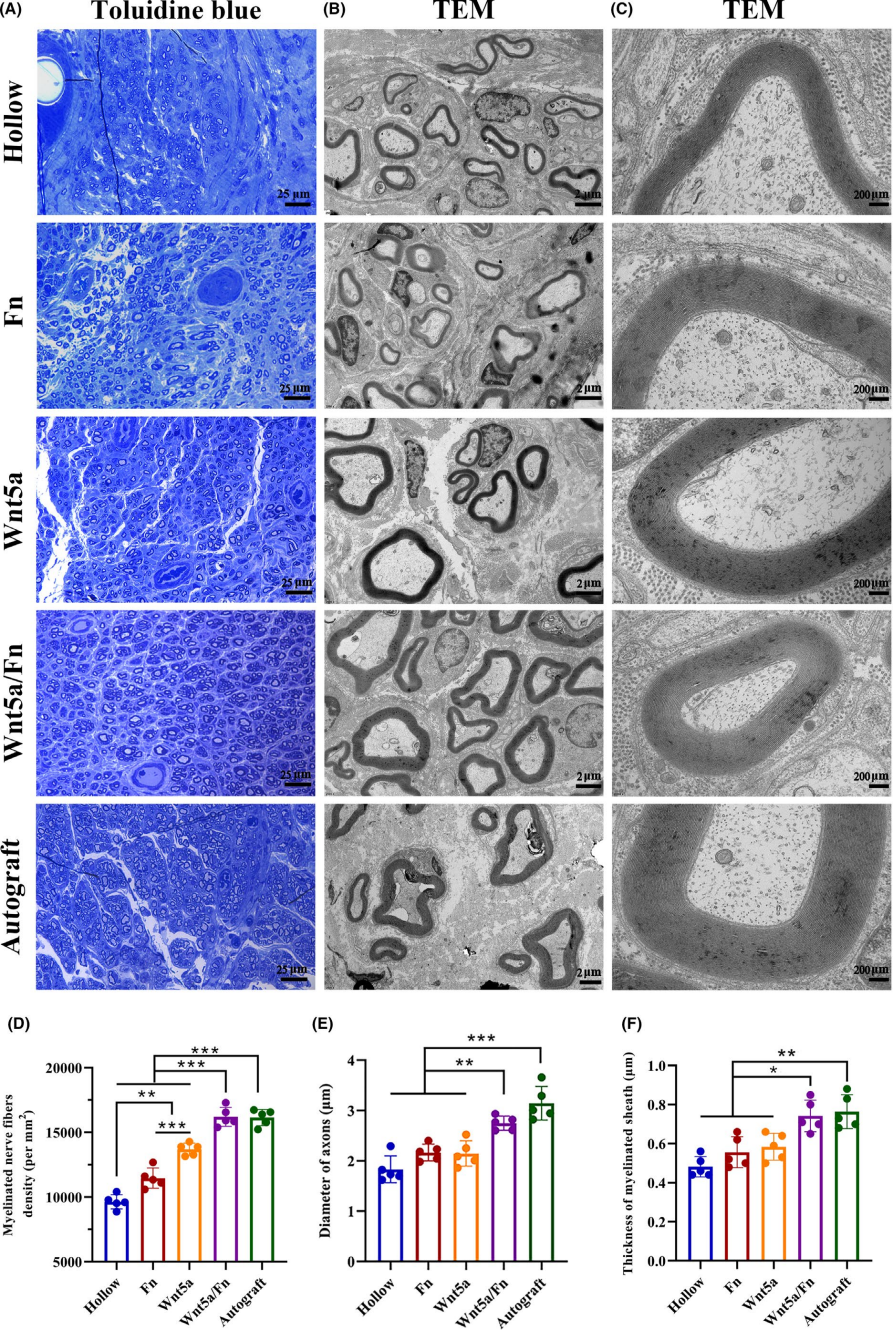
Histological evaluation of regenerated nerve at 12 weeks after the operation. (A) Representative views of toluidine blue staining of each group. Scale bar =25 μm. Original magnification is 400 ×. (B, C) Representative views TEM of each group. Scale bar =2 μm in (B) and 200 nm in (C). Original magnification is 2.5 kx in (B) and 20 kx in (C). (D–F) The myelin axons density, the diameter of axons, and the thickness of the myelin sheath of each group.*p < 0.05, **p < 0.01, ***p < 0.001. Data are expressed as the mean ±SD. TEM, transmission electron microscope; Fn, fibrin hydrogel group

The representative views of toluidine blue staining and TEM are shown in Figure 4. Toluidine blue staining is shown in Figure 4A. We found that the density of the myelinated nerve fibers in the Wnt5a/Fn and autograft groups was significantly greater than that in the hollow, Fn, and Wnt5a groups (*p* < 0.05). The density of myelinated nerve fibers in the hollow group was significantly lower than that in the Fn and Wnt5a groups (*p* < 0.05; Figure 4D). There was no statistical difference between the Wnt5a/Fn group and the autograft group in terms of myelinated nerve fiber density. The representative TEM images are shown in Figure 4B–C. The mean axonal diameters of the Wnt5a/Fn and autograft groups were significantly larger than those of the hollow, Fn, and Wnt5a groups (*p* < 0.05). The mean axon diameter in the autograft group was significantly greater than that in the Wnt5a/Fn group (Figure 4E). The mean thickness of the myelin sheath in the Wnt5a/Fn and autograft groups was significantly greater than that in the hollow, Fn, and Wnt5a groups, with no statistical difference between the Wnt5a/Fn and autograft groups (Figure 4F). The axon diameter and myelin thickness in the hollow group tended to be less than those in the Fn and Wnt5a groups (*p* > 0.05) (Figure 4E–F).

**FIGURE 5:**
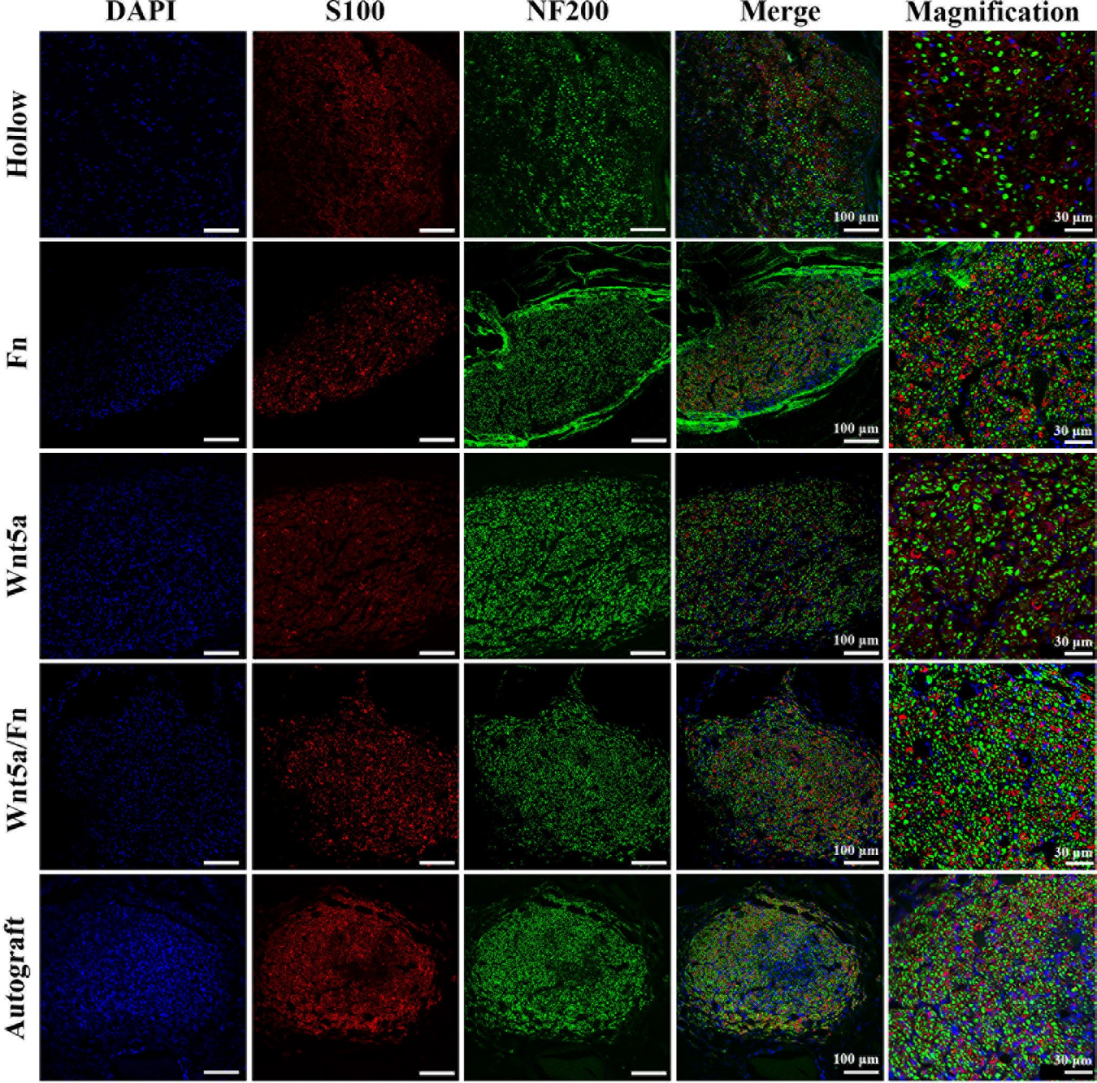
Representative cross‐sectional views of immunofluorescence staining of the regenerated nerve 12 weeks after the operation. The SCs were stained with S100 (red), axons with NF200 (green), nuclei with DAPI (blue). Scale bar =100 μm. Fn, fibrin hydrogel group

Representative cross‐sectional views of immunofluorescence staining of the regenerated nerve at 12 weeks after the operation are shown in Figure 5. We stained the SCs with S100 (red), axons with NF200 (green), and nuclei with DAPI (blue). Our observation revealed a greater axon density and myelination in the Wnt5a/Fn and autograft groups than those in the other three groups.

### 3.6 Angiogenesis in the regenerated nerve

**FIGURE 6:**
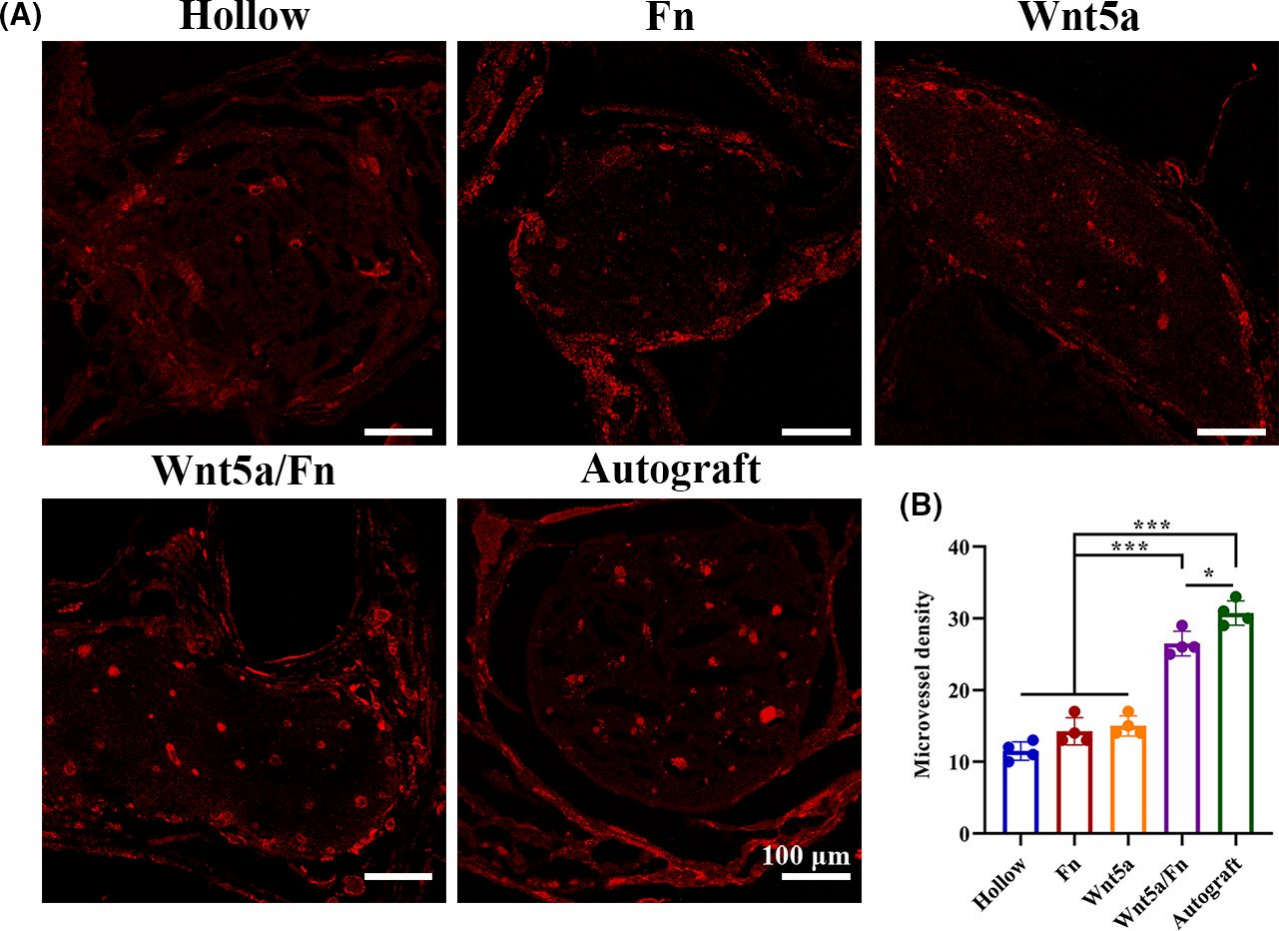
Angiogenesis evaluation 12 weeks after the operation. (A) Representative images of the cross section of regenerated nerve after microvessel immunofluorescence staining. Vascular endothelial cells were stained with CD31 (red). (B) Microvessel density (per cross section) in each group. Scale bar =100 μm. ***p < 0.001. Data are expressed as the mean ± SD

Immunofluorescence staining of the microvessels with regenerated nerve cross sections was performed 12 weeks after the operation (Figure 6A). Vascular endothelial cells were stained with CD31 (red). The microvessel density of the Wnt5a/Fn and autograft groups was superior to that of the hollow, Fn, and Wnt5a groups (*p* < 0.05; Figure 6B).

### 3.7 Gastrocnemius muscle recovery

**FIGURE 7:**
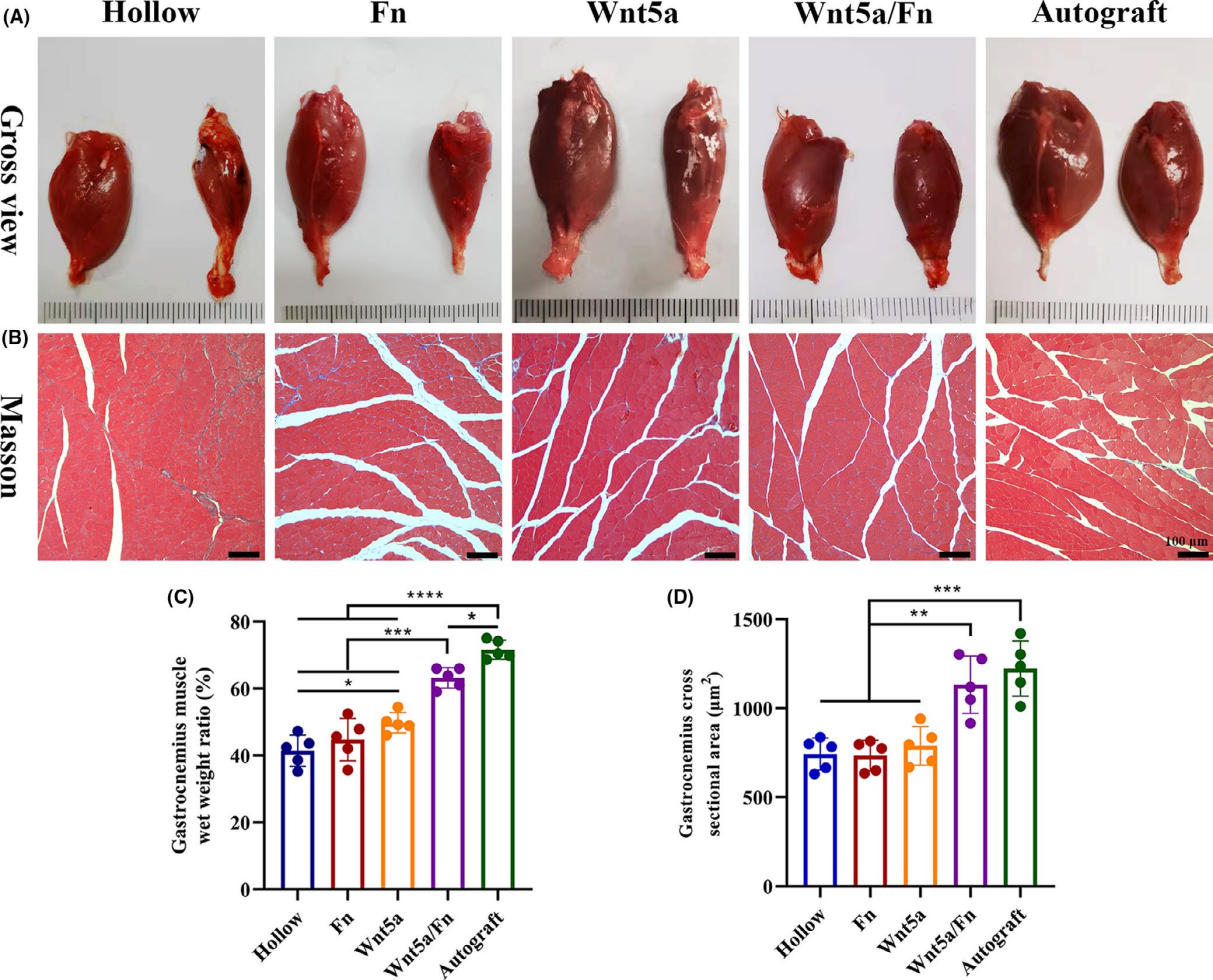
Gastrocnemius muscle recovery 12 weeks after the operation. (A) Representative gross views of gastrocnemius muscles of each group. The injured side was on the right. The normal side was on the left. (B) Representative images of Masson staining in each group. Original magnification is 100 ×. Scale bar =100 μm. (C) The wet weight ratio of the gastrocnemius (injured side/the normal side) of each group. (D) The mean cross‐sectional area of muscle fibers of each group. *p < 0.05, **p < 0.01, ***p < 0.001. Data are expressed as the mean ±SD. Fn, fibrin hydrogel group

The gastrocnemius muscles were collected from both sides and weighed, and Masson's trichrome staining was performed 12 weeks after the operation (Figure 7). We found that the injured side of gastrocnemius muscles in the hollow, Fn, and Wnt5a groups showed notable atrophy compared with the normal side. However, the difference in muscle volume between the injured and normal sides was not as obvious in the Wnt5a/Fn and autograft groups (Figure 7A). The wet weight ratio in the Wnt5a/Fn and autograft groups was significantly higher than that of the hollow, Fn, and Wnt5a groups (*p* < 0.05). Moreover, the wet weight ratio in the autograft group was higher than that of the Wnt5a/Fn group (*p* < 0.05), and that in the Wnt5a group was greater than that in the hollow group (*p* < 0.05; Figure 7C). Representative images of Masson's trichrome staining are shown in Figure 7B. We found that the hollow and Fn groups had more prominent blue‐stained collagen fibers. The mean gastrocnemius muscle cross‐sectional area of the Wnt5a/Fn and autograft groups was significantly greater than that of the hollow, Fn, and Wnt5a groups (*p* < 0.05; Figure 7D). There was no significant difference in the cross‐sectional area between the Wnt5a/Fn and autograft groups.

### 3.8 Gait analysis

**FIGURE 8:**
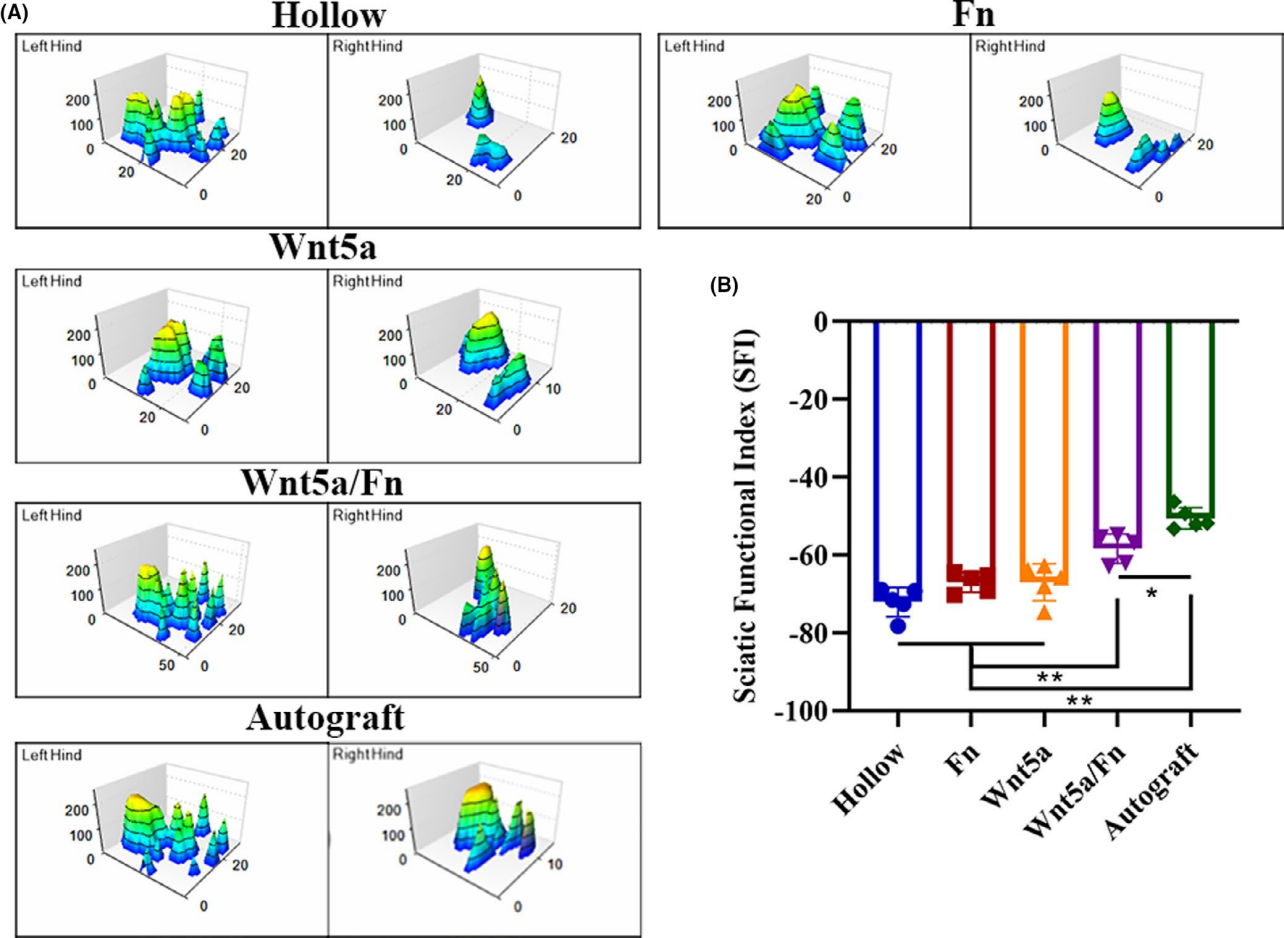
Gait analysis 12 weeks after the operation. (A) Representative three‐dimensional stress diagrams of each group. (B) SFI index of each group. *p < 0.05, **p < 0.01, ***p < 0.001. Data are expressed as the mean ±SD. Fn, fibrin hydrogel group. SFI, sciatic functional index

Gait analysis was performed 12 weeks after the surgery (Figure 8). According to the 3D stress diagrams (Figure 8A), we found that the plantar pressure, contact area, and the extension of the toes of the Wnt5a/Fn group were similar to those of the autograft group; these factors were worse in the hollow, Fn, and Wnt5a groups than in the Wnt5a/Fn and autograft groups. The SFI of the Wnt5a/Fn and autograft groups was significantly better than that of the hollow, Fn, and Wnt5a groups (*p* < 0.05), and that of the autograft group was better than that of the Wnt5a/Fn group (*p* < 0.05; Figure 8B).

## 4 DISCUSSION

Peripheral nerve injury is a significant clinical problem with a substantial impact on quality of life in patients.^2^, ^3^ Tissue‐engineered nerve grafts are easier to obtain; however, they remain less effective than autologous nerve transplantation. Therefore, more research on tissue engineering is needed, especially in the regenerative microenvironment.^1^, ^2^ To the best of our knowledge, this is the first study to examine the use of Wnt5a in repairing peripheral nerve defects.

In this study, we successfully fabricated a nerve conduit filled with Wnt5a‐loaded fibrin hydrogel. The fibrin hydrogel was synthesized by mixing with fibrinogen and thrombin at room temperature without requiring the addition of toxic cross‐linking agents, which indicated its practicability for clinical applications and good biocompatibility. The sustained‐release delivery of Wnt5a has a loading capacity of 73% and a release rate of 71.6%, which were superior to those of several previous studies on growth factor loaded hydrogel.^27^, ^28^ We revealed the fibrin hydrogel as a 3D porous structure with interconnected pores by SEM, which we speculate to be the structural basis of the sustained‐release effect. The 3D porous structure of fibrin hydrogel could also mimic the extracellular matrix, which is beneficial for cell adhesion, proliferation, and differentiation.^2^, ^29^ Several authors found that fibrin hydrogel could be used as a matrix for SC and dorsal root ganglion (DRG) cultures or as a VEGF‐releasing delivery system for the neural stem, demonstrating its biocompatibility with the neurocytes.^14^, ^15^, ^30^

We found that Wnt5a promoted in vitro SC proliferation. A previous study^10^ supported our findings by revealing that silencing Wnt5a expression by a GV248‐Wnt5a‐RNAi lentivirus could reduce SC proliferation. The promotion effect on SC proliferation may have therapeutic potential in PNI.^31^ Our study also indicated that Wnt5a could promote the gene expression of VEGF, NGF, and CNTF, as well as the protein secretion of VEGF and NGF. We hypothesized that SC and macrophage are cellular targets of Wnt5a‐induced secretion. On the one hand, we found that Wnt5a can promote SCs release VEGF, NGF, and CNTF in vitro. In the pathophysiological process of peripheral nerve regeneration, several neurotrophic factors are mainly secreted by SC, such as GDNF and BDNF.^32^, ^33^, ^34^ On the other hand, we also revealed that Wnt5a sustained‐release system can promote microvessel density in the regenerated nerve. Macrophage plays an important role in inducing blood vessel formation via VEGF secretion in peripheral nerve regeneration,^13^ which may be one of the cellular targets of Wnt5a. VEGF is a crucial factor for inducing angiogenesis and guiding SC migration and axonal extension in peripheral nerve regeneration.^13^ VEGF can also directly promote dose‐dependent SC proliferation and axonal outgrowth.^35^, ^36^ Lee et al.^14^ fabricated a VEGF‐loaded fibrin gel that facilitated the proliferation and migration of neural stem cells.

Nerve growth factor is a prominent neurotrophin in peripheral nerve repair and is commonly used as a lumen filler in the tissue‐engineered nerve conduit, which promotes sensory neuron regeneration.^2^, ^37^, ^38^ Wnt5a is closely related to the NGF signaling pathway. Bodmer et al^9^ found that Wnt5a is a key downstream protein of NGF signaling in the sympathetic nervous system, prompting NGF‐dependent axonal branching and growth by stimulating calpain activity.

We found that Wnt5a can promote the release of VEGF and NGF. Several studies have found that the delivery system of multiple NTFs was more effective than a single NTF delivery system.^39^, ^40^, ^41^ The combined CNTF and basic fibroblast growth factor (bFGF) group were superior to the single CNTF or bFGF group in terms of the number of regenerated nerves, myelination, and motor function recovery in facial nerve injury.^39^ A collagen nerve conduit loaded with glial cell line‐derived neurotrophic factor (GDNF) and NGF was more effective than that loaded with GDNF alone in terms of SC migration and axonal growth in the 10‐mm rat sciatic nerve injury model.^40^ Furthermore, Lu et al^41^ found that the brain‐derived neurotrophic factor (BDNF) and VEGF‐mimetic peptide could promote better peripheral nerve regeneration than the single BDNF or VEGF‐mimetic peptide group resulting from the synergistic effect of neurotrophic and pro‐angiogenic factors. These results indicated that a multiple NTF delivery system combining vascularization and neurotrophic growth cues could provide additional advantages due to better neurovascular microenvironment reconstruction when compared to single NTF delivery systems. From this perspective, we consider Wnt5a‐loaded fibrin hydrogel as a multiple NTF delivery system with the synergistic effect of vascularization and nerve regeneration because Wnt5a can promote the release of various NTFs, such as VEGF, NGF, and CNTF, which indicated that it may be superior to previous single NTF delivery systems.^37^, ^42^ Additionally, we used a single signaling molecule, rather than multiple neurotrophic factors to obtain multiple neurotrophin secretions, which is a more convenient drug delivery approach compared with traditional approaches.^41^, ^43^

The animal experimental results showed that the Wnt5a solution group was superior to the hollow group in terms of myelinated nerve fiber density, CMAP amplitude, and wet weight ratio of the gastrocnemius muscle. Moreover, the nerve repair effect of the Wnt5a/Fn group was similar to that of the autograft group in terms of axon diameter, myelinated sheath thickness, axon density, microvessel density, CMAP latency, and mean gastrocnemius muscle cross‐sectional area. However, the autograft group was superior in terms of SFI, wet weight ratio of the gastrocnemius muscle, and CMAP amplitude compared with the Wnt5a/Fn group, which was similar to previous studies.^44^, ^45^ The SFI and wet weight ratio of the gastrocnemius muscle are crucial indicators for motor function recovery,^21^, ^25^ and the CMAP amplitude is associated with the number of reinnervated muscle fibers.^46^ Thus, we speculate that the repair effect of the Wnt5a/Fn group remains inferior to that of the autograft group, which mainly for the following reasons. First, although fibrin hydrogel could be used as a matrix for SC and DRG cultures, it could not fully mimic the extracellular matrix of autologous nerve tissues. Second, despite Wnt5a can promote the release of various NTFs, it could not fully simulate the types, concentrations, and release profiles of NTFs in autologous nerves, which is essential for PNI regeneration.^37^, ^43^

This study has several limitations. First, we showed that Wnt5a can promote SC proliferation and neurotrophic factor secretion in vitro. The in vivo experiment revealed that the Wnt5a sustained‐release system can promote the repair of PNI. However, we did not investigated the mechanism of Wnt5a on peripheral nerve regeneration in vivo, which may be complicated and implicated in multiple aspects of peripheral nerve regeneration. We consider this as an important direction for future research. Second, sex‐related differences were observed in cerebral vascular regulation, and metabolism,^47^ vulnerability to ischemic injury,^48^ and immune cell responses in rats.^49^ Although most previous studies on peripheral nerve defect used single‐sex rats,^13^, ^20^, ^25^ there is still a risk of bias in this study because all rats included were female.

In summary, we fabricated a nerve conduit filled with Wnt5a‐loaded fibrin hydrogel, which was a sustained‐release system, to repair a 10‐mm rat sciatic nerve defect. In vitro, Wnt5a could promote SC proliferation and the gene expression of VEGF, NGF, and CNTF, as well as the protein secretion of VEGF and NGF. In vivo, the Wnt5a/Fn group was superior to the hollow, fibrin hydrogel, and Wnt5a groups in terms of axonal growth, myelination, electrophysiological recovery, target organ innervation, and motor function recovery at 12 weeks after the operation. This study demonstrated the promotional effect of a nerve conduit filled with Wnt5a‐loaded fibrin hydrogels on peripheral nerve defects for the first time. The mechanism for this may be the secretion of multiple NTFs combining vascularization and neurotrophic growth cues. Future studies should focus on the molecular mechanisms of Wnt5a in peripheral nerve regeneration.

## CONFLICTS OF INTEREST

The authors declare no conflict of interest.

## AUTHOR CONTRIBUTIONS

Yi‐jun Liu contributed to investigation, methodology, data curation, software, and validation. Xiao‐Feng Chen contributed to validation and writing—original draft. Li‐ling Zhou contributed to material mechanic experiment and writing—original draft. Rao Feng contributed to project administration, resources, data curation, formal analysis and validation. Dian‐Ying Zhang contributed to supervision and funding acquisition. Yan‐hua Wang contributed to supervision, writing—review, and funding acquisition.

## Data Availability

The data are available from the corresponding author upon reasonable request.
